# Artificial Intelligence Prediction Model for the Cost and Mortality of Renal Replacement Therapy in Aged and Super-Aged Populations in Taiwan

**DOI:** 10.3390/jcm8070995

**Published:** 2019-07-09

**Authors:** Shih-Yi Lin, Meng-Hsuen Hsieh, Cheng-Li Lin, Meng-Ju Hsieh, Wu-Huei Hsu, Cheng-Chieh Lin, Chung Y. Hsu, Chia-Hung Kao

**Affiliations:** 1 Graduate Institute of Biomedical Sciences, China Medical University, Taichung 404, Taiwan; 2 Division of Nephrology and Kidney Institute, China Medical University Hospital, Taichung 404, Taiwan; 3 Department of Electrical Engineering and Computer Sciences, University of California, Berkeley, CA 94720, USA; 4 Management Office for Health Data, China Medical University Hospital, Taichung 404, Taiwan; 5 College of Medicine, China Medical University, Taichung 404, Taiwan; 6 Department of Medicine, Poznan University of Medical Sciences, 061 Poznan, Poland; 7 Division of Pulmonary and Critical Care Medicine, China Medical University Hospital and China Medical University, Taichung 404, Taiwan; 8 Department of Family Medicine, China Medical University Hospital, Taichung 404, Taiwan; 9 Department of Nuclear Medicine and PET Center, China Medical University Hospital, Taichung 404, Taiwan; 10 Department of Bioinformatics and Medical Engineering, Asia University, Taichung 404, Taiwan

**Keywords:** end-stage renal disease (ESRD), dialysis, artificial intelligence modeling, National Health Insurance Research Database (NHIRD)

## Abstract

Background: Prognosis of the aged population requiring maintenance dialysis has been reportedly poor. We aimed to develop prediction models for one-year cost and one-year mortality in aged individuals requiring dialysis to assist decision-making for deciding whether aged people should receive dialysis or not. Methods: We used data from the National Health Insurance Research Database (NHIRD). We identified patients first enrolled in the NHIRD from 2000–2011 for end-stage renal disease (ESRD) who underwent regular dialysis. A total of 48,153 Patients with ESRD aged ≥65 years with complete age and sex information were included in the ESRD cohort. The total medical cost per patient (measured in US dollars) within one year after ESRD diagnosis was our study’s main outcome variable. We were also concerned with mortality as another outcome. In this study, we compared the performance of the random forest prediction model and of the artificial neural network prediction model for predicting patient cost and mortality. Results: In the cost regression model, the random forest model outperforms the artificial neural network according to the mean squared error and mean absolute error. In the mortality classification model, the receiver operating characteristic (ROC) curves of both models were significantly better than the null hypothesis area of 0.5, and random forest model outperformed the artificial neural network. Random forest model outperforms the artificial neural network models achieved similar performance in the test set across all data. Conclusions: Applying artificial intelligence modeling could help to provide reliable information about one-year outcomes following dialysis in the aged and super-aged populations; those with cancer, alcohol-related disease, stroke, chronic obstructive pulmonary disease (COPD), previous hip fracture, osteoporosis, dementia, and previous respiratory failure had higher medical costs and a high mortality rate.

## 1. Introduction

Improving care for patients with chronic kidney disease (CKD) and associated comorbidities might lead to better outcomes and slows the progression of CKD [1]. Therefore, demand has been increasing for dialysis among older patients [2,3]. Geriatric dialysis or dialysis in later life is increasingly relevant [4]. According to some reports, dialysis treatment increases the risk of frailty [5,6], functional impairment [7], cognition decline [8], and accidental falls [9] among older adults, as well as increasing medical costs and mortality rates [10]. Awareness is growing regarding appropriate dialysis care for such individuals.

To ensure better life quality, current guidelines have prompted shared decision-making concerning dialysis initiation, especially for older adults [11,12,13]. In shared decision-making, the clinician offers options and describes the risks and benefits of dialysis and renal replacement therapy, and patients express their preferences and values [14,15]. Therefore, clinicians should identify the factors that carry risks of mortality or of increased caregiving constraints and medical costs after older patients have entered dialysis treatment. Studies have identified several mortality risk factors in older adults undergoing dialysis [16,17,18,19].

Tamura et al. showed that most institutionalized older adults died after one year and suffered deteriorating quality of life after initiation of dialysis [20]. By contrast, Derrett et al. investigated older adults being cared for at home and discovered that age was not the major determining factor for mortality [21]. Controversy regarding the initiation of dialysis in older adults has persisted. With Taiwan’s population aging, the cutoff age of 65 years that is used for the identification of conventional mortality risk factors might not be suitable for the super-aged who are aged more than 80 years. Further, mortality risk for dialysis patients is reported to be highest in the first year; up to 20.4% [22]. It is also reported that 70.9% of all deaths in Taiwan are attributed to the population aged 65 and over in 2016 [23]. Therefore, mortality and medical costs are two major concerns in decision-making for patients and family members regarding dialysis for older adults. A prediction model that considers potential differences between aged and super-aged patients requiring dialysis in terms of mortality and costs is required to meet the demands of clinicians. In this study, we applied two prediction models, a random forest prediction model and an artificial neural network model, and adopted the National Health Insurance Research Database (NHIRD). We aimed to (1) compare the performance of the random forest and artificial neural network prediction models and (2) select a suitable model for predicting one-year mortality and costs for elderly patients.

## 2. Method

### 2.1. Data Source and Sampled Participants

The study was approved by the Research Ethics Committee of China Medical University and Hospital in Taiwan (CMUH104-REC2-115-CR3). The National Health Insurance program in Taiwan was implemented in 1995 and provides comprehensive medical care, including ambulatory and inpatient care, to nearly 99% of Taiwan’s population, which is approximately 23 million people. For this study, we used data from the National Health Insurance Research Database (NHIRD). We identified patients first listed in the NHIRD between 2000 and 2011 for end-stage renal disease (ESRD) who had undergone regular dialysis (ICD-9-CM Code 585). Patients with ESRD aged ≥65 years with complete information about age and sex were included in the ESRD cohort. Because dialysis patients usually had many comorbidities, we used total costs to reflect the real medical costs (measured in US dollars) within one year after ESRD diagnosis as our main outcome variable. The total medical costs per patient within one year included dialysis, admission, drug, fistula, catheters, and all other medical services, such as cardiac catheterization and gastroduodenal endoscopy, etc. We were also interested in mortality as another outcome.

### 2.2. Data Availability Statement

The dataset used in this study is held by the Taiwan Ministry of Health and Welfare (MOHW). The Ministry of Health and Welfare must approve our application to access this data. Any researcher interested in accessing this dataset can submit an application form to the Ministry of Health and Welfare (MOHW) requesting access. Please contact the staff of MOHW. All relevant data are within the paper.

### 2.3. Ethics Statement

The NHIRD encrypts patient personal information to protect privacy and provides researchers with anonymous identification numbers associated with relevant claims information, including sex, date of birth, medical services received, and prescriptions. Therefore, patient consent is not required to access the NHIRD. This study was approved to fulfill the condition for exemption by the Institutional Review Board (IRB) of China Medical University (CMUH-104-REC2-115-CR2). The IRB also specifically waived the consent requirement.

### 2.4. Variables of Interest

The sociodemographic variables used included age, sex, urbanization level, and occupation. Medical care was analyzed within one year and included the total duration of hospitalization in days and the frequency of medical visits. The NHRI stratified all city districts and townships in Taiwan into seven urbanization levels, based on population density (people/km^2^), proportion of residents with higher education, elderly and agricultural population, and the number of physicians per 100,000 people in each area. Level 1 represented areas with a higher population density and socioeconomic status, and Level 7 represented the lowest. Because few people lived in more rural areas of levels 4–7, our study grouped these areas into the group of 4 levels.

Baseline comorbidities included diabetes, hypertension, hyperlipidemia, liver disease and cirrhosis, coronary artery disease, obesity, cancer, alcohol-related disease, cirrhosis, stroke, GI bleeding, COPD, previous hip fracture, osteoporosis, dementia, previous herpes, and previous respiratory failure.

### 2.5. Training Dataset Development

The original raw data contained features including age, sex, urbanization level, occupation, and comorbidities. The urbanization level and occupation of each subject was label encoded in the raw dataset. Four urbanization levels and four occupation levels were used. These categories were one-hot encoded, creating eight additional features. In total, there were 33 features in the dataset for predicting patient mortality and 34 features for predicting patient cost. Patient mortality was used to predict patient cost, but cost was not used to predict mortality.

Each data point was randomly allocated to training and testing sets at a ratio of 90:10. For the continuous features in these sets, unity-based normalization and standardization was applied based on the mean and variance of the training set.

### 2.6. Algorithm Training

#### 2.6.1. Cost Regression Model

Before the random forest regression model was trained, the optimal depth of each decision tree in the random forest was determined. This was done by plotting the mean squared error and mean absolute error against the maximum depth of the model. The optimal depth was determined by the depth at which the metrics for the training and testing sets begin to diverge.

After the optimal depth was determined, the random forest regression model was trained. Twenty decision tree predictors were used in the model. The cost regression model was evaluated using the mean squared error and mean absolute error metrics. The mean squared error was used to measure the split quality by minimizing L_2_ loss. The minimum samples per split and minimum samples per leaf were set to two and one respectively. The lower the error values, the better the regression models perform.

The artificial neural network regression model was a deep neural network with three hidden layers. The input layer had 33 dimensions, each hidden layer had 17 dimensions, and the output layer had one dimension, which represented the predictive value. Each hidden layer used the scaled exponential linear unit activation function [24], and the output layer did not have an activation function. The model used the mean squared error as loss and was optimized with the Adam optimizer [25].

#### 2.6.2. Mortality Classification Model

The random forest classification model was trained with 20 decision tree predictors with maximum depths of 17. Gini impurity was used to measure the split quality. The minimum number of samples per split and the minimum number of samples per leaf were also set to two and one, respectively.

The artificial neural network classification model was a deep neural network. The architecture of the model is similar to the regression model with some differences. The model used the cross-entropy loss function. The output layer used the softmax activation function and had two dimensions, each of which represented an outcome: Survival or death.

The random forest models were developed using Python (version 3.7.0) with the scikit-learn framework (version 0.19.2) [26]. The artificial neural network models were developed using Python (version 3.7.0) with the Tensorflow Library (version 1.11.0) [27].

#### 2.6.3. Evaluation of Models

The regression models were evaluated using the mean squared error and mean absolute error across the training set, testing set, and all data. The classification models were evaluated using k-fold cross-validation accuracy (k = 10); the confusion matrix metrics of recall (sensitivity), precision (positive predictive value), and F1 (harmonic mean between recall and precision); and the area under the receiver operating characteristic (ROC) curve. The ROC curves were generated based on prediction probabilities.

**Figure 1:**
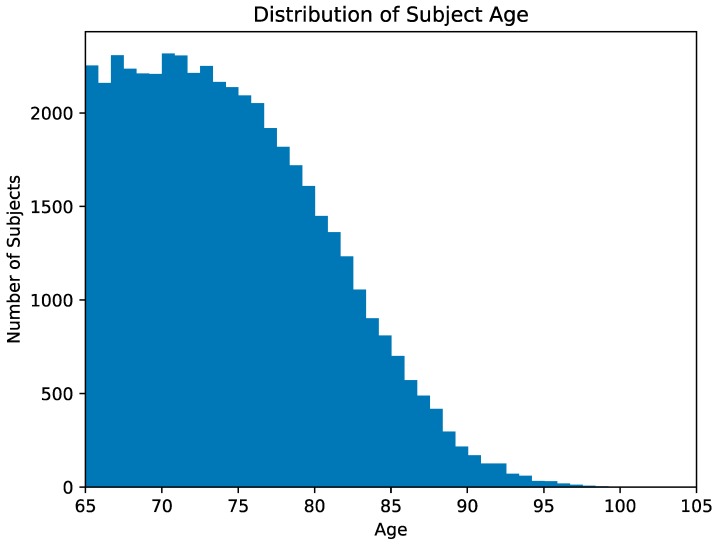
Presents a histogram of subjects classified by age.

In addition, additional recall, precision, and F1 values for the classification models were calculated for patient cohorts classified by age. The patients were separated into six age groups: <70, 70–75, 75–80, 80–85, 85–90, and >90. Figure 1 presents a histogram of subjects classified by age.

### 2.7. Statistical Analyses of Demographic Features

Proportions for categorical variables, and the median ±interquartile range (IQR) for continuous variables were presented for demographic data. Differences in sociodemographic distributions and baseline comorbidity between survival and death in patients ESRD were examined using the chi-squared test for categorical variables and the Student’s *t*-test for mean age, total duration of hospitalization, and frequency of medical visits. Data management was undertaken using SAS 9.4 software (SAS Institute; Cary, NC, USA). All P-values were two-tailed, and *p*-values of <0.05 were considered significant.

## 3. Results

### 3.1. Demographic Features of Patients

Table 1 reveals that a total of 48,153 ESRD patients were identified as the study participants. These patients also had a high prevalence of hypertension, coronary artery disease (CAD), gastrointestinal (GI) bleeding, diabetes, previous hip fracture, osteoporosis, dementia, and hyperlipidemia. The mean total duration of hospitalization was seven days (IQR = 0–26). The mean frequency of medical visits within one year was 35 (IQR = 21–51).

Table 2 reveals that compared with patients who survived, those who died were older (77.0 ± 6.79 years vs. 74.1 ± 6.08); patients who died also had a higher likelihood of experiencing more than 5 comorbidities and a higher prevalence of diabetes, cancer, alcohol-related disease, stroke, COPD, previous hip fracture, osteoporosis, dementia, and previous respiratory failure.

### 3.2. Evaluation of Prediction Models

#### 3.2.1. Cost Regression Model

**Figure 2:**
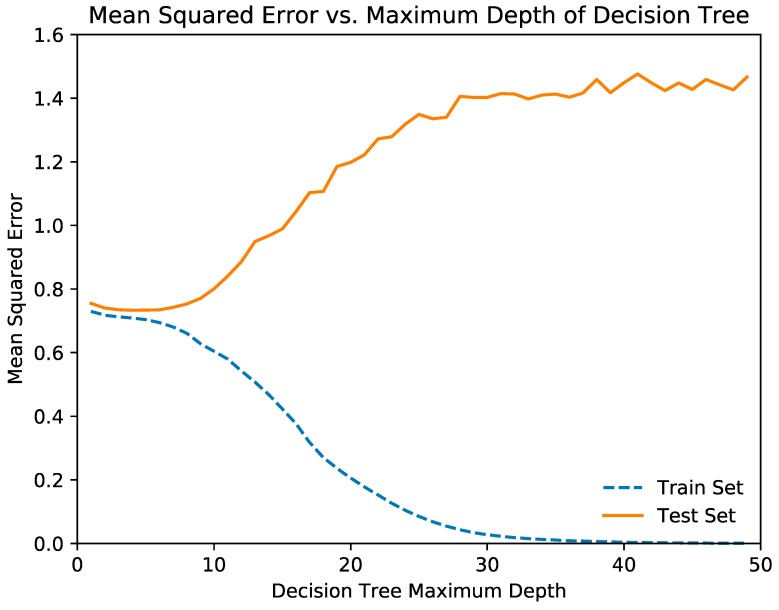
The relationships of the decision tree depth with the mean squared error.

**Figure 3:**
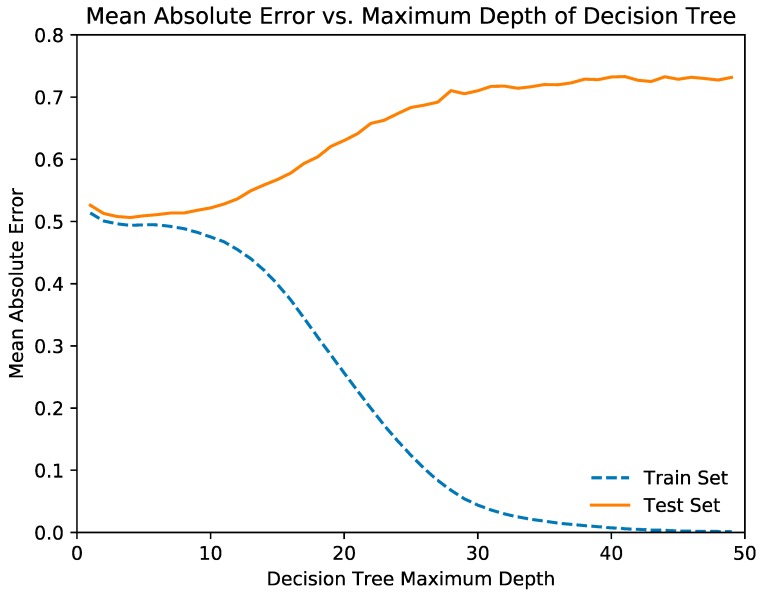
Illustrates the relationships of the decision tree depth with mean absolute error.

Figure 2 and Figure 3 illustrate the relationships of the decision tree depth with the mean squared error and mean absolute error, respectively. From the graph, we determined that the optimal maximum decision tree depth was 7, and we trained the decision tree model with this depth. Table 3 and Table 4 show the mean squared error and mean absolute error of the random forest and neural network regression models, respectively.

The metrics for the test set reveal that the two models are able to generalize predictions relative to performance in the training set. The random forest model outperforms the artificial neural network according to the mean squared error and mean absolute error.

#### 3.2.2. Mortality Classification Model

**Figure 4:**
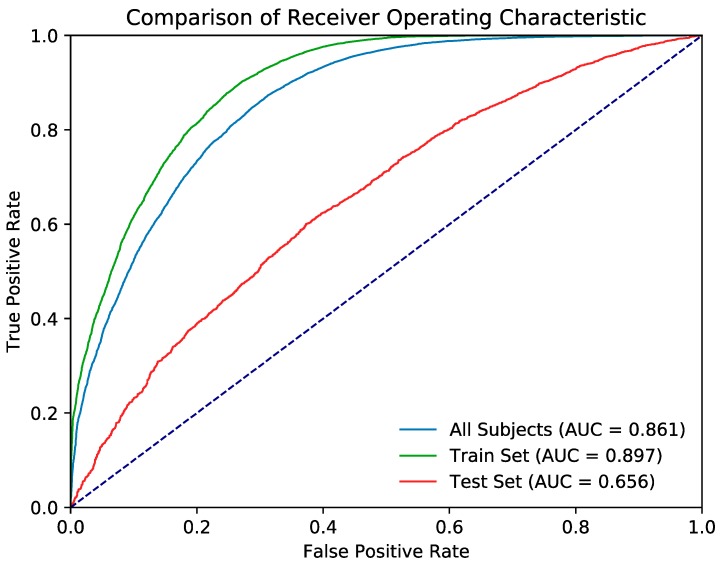
Presents the receiver operating curve (ROC) of the evaluation metrics of the random forest classification model.

**Figure 5:**
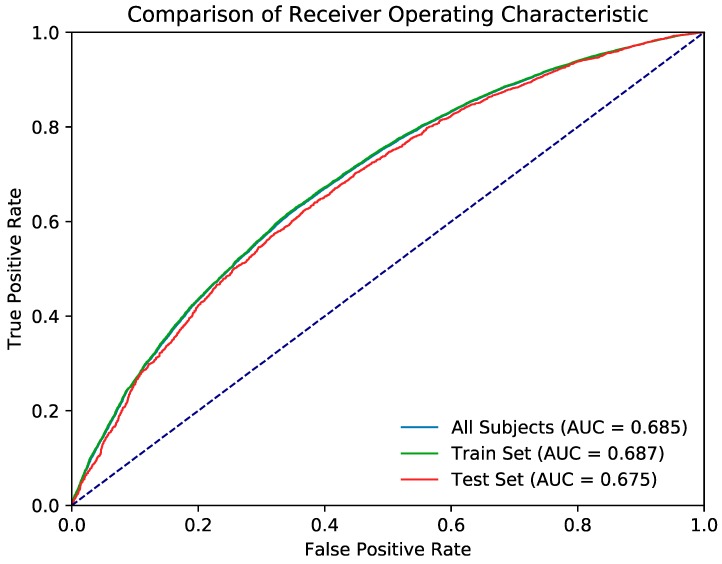
The ROC curve of the evaluation metrics of the artificial neural network classification model.

Table 5 provides the evaluation metrics of the random forest classification model, and Figure 4 presents the ROC curve. The k-fold cross-validation accuracy (k = 10) of the random forest model was 0.745. Table 6 provides the evaluation metrics of the artificial neural network classification model, and Figure 5 shows the ROC curve. The k-fold cross-validation accuracy of the neural network model was also 0.745.

The ROC curves of both models were significantly better than the null hypothesis area of 0.5. Although the random forest model outperformed the artificial neural network in the train set and across all data, both models achieved similar performance in the test set.

Table 7 lists the patient mortality rates within for the various age cohorts, and Table 8 and Table 9 provides the metrics of the models for these groups. The random forest and artificial neural network classification models yielded the highest accuracies with the cohort of patients aged <70 years of age.

## 4. Discussion

Our study provided a prediction model for one-year mortality and costs for older patients undergoing dialysis in Taiwan. Shin et al. demonstrated that, in older patients, dialysis may be associated with increased mortality risk and increased healthcare cost compared with conservative care [28]. Because of this, mortality rates are higher among older adults, and the costs are high after dialysis is commenced. Therefore, this information would be inadequate for assisting decision-making concerning whether older patients should receive dialysis. In addition to providing prediction models, this study identified that occupation, duration of hospitalization in days, and prevalence of cancer, alcohol-related disease, stroke, COPD, previous hip fracture, osteoporosis, dementia, and previous respiratory failure were associated with increasing medical expenditures. Furthermore, diabetes, cancer, alcohol-related disease, stroke, COPD, historical hip fracture, osteoporosis, dementia, and previous respiratory failure were associated with increasing mortality. Thus, if older patients have cancer, alcohol-related disease, COPD, previous hip fracture, osteoporosis, dementia, or previous respiratory failure, we would be more likely to inform patients and their family members that increasing mortality and medical costs were anticipated. Our results seemed somehow expected since the more aged and the more comorbidities a given population has, the higher is the mortality risk and the more expensive the health care is. However, these results were provided through analytic methods and machine learning. Our data could provide convincing information for helping clinicians to illustrate the prognosis of aged population requiring dialysis. Our data also could help family members and aged patients to decide whether receiving dialysis or not, especially for those more aged and more comorbidities. Li et al. revealed falls as an independent risk factor for mortality among older adults [16]. However, one identifiable risk factor alone might not suffice for making decisions regarding dialysis. Foote et al. concluded that a body mass index <18.5, numerous comorbidities, late referral, peritoneal dialysis as intended modality, and unprepared access were mortality risk factors [17]. Furthermore, Thamer et al. used 14 risk factors, of which eight factors were considered in our study, to establish a comprehensive risk score for older patients undergoing dialysis [18]. Score points would tend to be arbitrary, but several risk factors that may be critical for older adults—including orthopedic problems and stroke—were not considered [18]. The AUC was 0.72 in the study of Thamer et al., which might be considered imprecise in the context of medical decision-making [18]. Couchoud et al. used nine risk factors, of which some overlapped with this study and the study of Thamer et al [18,29]. However, failing to consider alcoholism, osteoporosis, hip fracture, and liver disease would limit the application of their findings to older Taiwanese adults. Cohen et al. also developed a risk model in assessing six-month mortality [30]. Although this study was comprehensive, the small population size limited the applicability for the model [30].

Similar to the results of other studies, [18,29,31] our study demonstrated that obesity reduced the risk of mortality and medical expenditures. However, contrary to the findings of other studies [18,29,30] our study did not directly associate risk of mortality and increasing medical expenditures with numbers of comorbidities. Explanations for this could relate to our studies incorporation of comorbidities as possible, and some of our factors conferred protection. Our study might prompt awareness among clinicians that conservative care is not recommended only based on the number of comorbidities.

Artificial neural networks have been successfully applied in medical fields [32,33,34,35,36]. The random forest prediction model is a multivariate prediction model that combines several decision trees to determine the most likely output [37]. The artificial neural network model is a multivariate prediction model composed of connected units, or neurons, with computations inspired by biological systems [38]. Per both the mean squared error (MSE) and mean absolute error (MAE) metrics, the random forest model outperformed the artificial neural network in cost prediction for all cohorts. Per the F_1_ metric, the random forest model performed better than the artificial neural network in predicting mortality rates for cohorts older than 85 years, and the artificial neural network performed better for cohorts younger than 85 years. We considered possible explanations for the superiority of the random forest model, such as the complexity of underlying diseases among older patients undergoing dialysis population.

Several limitations of this study are noteworthy: First, this study had no external validation. Second, because the majority of participants in this study were Taiwanese, our model should be applied to other populations with caution, and further validation with different populations is required to deploy our model at a widespread scale. Third, detailed information about clinical frailty scales [39], routine activities [40], body mass index, glucose levels, albumin levels, hemoglobin levels, calcium levels, ferritin levels, C-reactive protein levels, promptness of patient referral, left ventricular ejection fraction, medications (anticoagulants, immunosuppressant, etc.), smoking status, net ultrafiltration, and blood pressure were unavailable in the NHIRD. Therefore, our model would be hard to be compared directly with the REIN score and Aroscore. However, if achieved it would be another advantage of our prediction model since the information and the variables we provided may be available in most countries’ database. Fourth, the absence of precise assessment for depression and quality of life must be recognized. Thus, our prediction model could help determine patients’ survival and medical costs but does not guarantee the quality of life of older patients undergoing dialysis. Fifth, this study investigated only older patients undergoing dialysis; thus, those who had refused dialysis were not analyzed in this study. It would be one of the possible explanations why our model is less precise in patients who were older. Finally, information about the location of these patients where they lived in nursing home, senior living, independent living, or home are unavailable. Therefore, the cost calculated and provided in our model is the total medical cost in the first year of dialysis rather than the total care cost in the first year of dialysis.

## 5. Conclusions

Our study demonstrates that random forest modeling can provide reliable information about one-year outcomes following dialysis in the aged and super-aged populations, especially for individuals with major comorbidities. Our models are believed to provide more information to assist older patients and their family members in deciding whether to start dialysis.

## Figures and Tables

**Table 1:** Demographic characteristics and comorbidities by median medical expenditures in US$ within one year after end-stage renal disease (ESRD) entrance.

Variable	One-Year Medical Expenditures, *n* = 48,153
	*n* (%)
**Age, year**	
65–79	26,732 (55.5)
80+	21,421 (44.5)
**Median (IQR, interquartile range)**	74.0 (69.5, 79.0)
**Sex**	
Female	25,994 (54.0)
Male	22,159 (46.0)
**Urbanization level ^&^**	
1 (Highest urbanization)	11,708 (24.3)
2	12,842 (26.7)
3	8162 (15441)
4(Lowest urbanization)	15,441 (32.1)
**Occupation**	
Housekeeping	21,924 (45.5)
White collar	931 (1.93)
Blue collar	15,013 (31.2)
Others ^‡^	10,285 (21.4)
One-year expenditures	
**Median** (25th and 75th percentile)	US$20,846 (US$12,468–US$22,802)
**Total duration of hospitalization in days stay within one year after ESRD diagnosis** **Median (IQR, interquartile range)**	7 (0–26)
**Frequency of medical visits within one year after ESRD diagnosis** **Median (IQR, interquartile range)**	35 (21–51)
**Comorbidity**	
Diabetes	25,759 (53.5)
Hypertension	45,371 (94.2)
Hyperlipidemia	23,329 (48.5)
Liver disease and cirrhosis	9982 (20.7)
Coronary artery disease	28,396 (59.0)
Obesity	433 (0.90)
Cancer	4497 (9.34)
Alcohol–related disease	1232 (2.56)
Cirrhosis	11,927 (24.8)
Stroke	12,469 (25.9)
GI bleeding	26,775 (55.6)
COPD	13,818 (28.7)
Previous hip fracture	8245 (17.1)
Osteoporosis	9717 (20.2)
Dementia	3198 (6.64)
Previous herpes	2558 (5.31)
Previous respiratory failure	2560 (5.32)
Number of comorbidities	
≤5	22,884 (47.5)
>5	25,269 (52.5)

Data are presented as the number of subjects in each group with percentages given in parentheses or mean with standard deviation given in parentheses. ^&^ Urbanization was categorized into four levels according to the population density of the residential area, with Level 1 the most urbanized and Level 4 the least urbanized. ^‡^ Other occupations included primarily retired, unemployed, or low income populations. ESRD, end stage renal disease.

**Table 2:** Demographic characteristics and comorbidities between patients with one-year mortality or without one-year mortality after ESRD entrance; Individual odds ratio of baseline demographic and comorbidities for one-year mortality after ESRD entrance.

Variable	One-Year Mortality after ESRD Entrance		Odds Ratio (95% CI)	*p*-Value
	No, *n* = 37742	Yes, *n* = 10411		
	*n* (%)	*n* (%)		
**Age, year**				<0.001
65–79	22447(59.5)	4285(41.2)	1.00	
80+	15295(40.5)	6126(58.8)	2.10(2.01, 2.19)	
Mean ± SD ^†^	74.1(6.08)	77.0(6.79)		<0.001
**Sex**				<0.001
Female	20702(54.9)	5292(50.8)	1.00	
Male	17040(45.2)	5119(49.2)	1.18(1.13, 1.23)	
**Urbanization level ^&^**				0.66
1 (Highest urbanization)	9155(24.3)	2553(24.5)	1.03(0.97, 1.09)	
2	10051(26.6)	2791(26.8)	1.03(0.97, 1.09)	
3	6381(16.9)	1781(17.1)	1.03(0.97, 1.10)	
4 (Lowest urbanization)	12155(32.2)	3286(31.6)	1.00	
**Occupation**				<0.001
Housekeeping	17279(45.8)	4645(44.6)	1.02(0.97, 1.07)	
White collar	744(1.97)	187(1.80)	0.95(0.81, 1.13)	
Blue collar	11881(31.5)	3132(30.1)	1.00	
Others ^‡^	7838(20.8)	2447(23.5)	1.18(1.12, 1.26)	
**Comorbidity**				
Diabetes	19965(52.9)	5794(55.7)	1.12(1.07, 1.17)	<0.001
Hypertension	35660(94.5)	9711(93.3)	0.81(0.74, 0.89)	<0.001
Hyperlipidemia	18768(49.7)	4561(43.8)	0.79(0.76, 0.82)	<0.001
Liver disease and cirrhosis	7853(20.8)	2129(20.5)	0.98(0.93, 1.03)	0.43
Coronary artery disease	22294(59.1)	6102(58.6)	0.98(0.94, 1.03)	0.40
Obesity *	371(0.98)	62(0.60)	0.60(0.46, 0.79)	<0.001
Cancer	3146(8.14)	1720(12.9)	1.71(1.60, 1.83)	<0.001
Alcohol–related disease	902(2.39)	330(3.17)	1.34(1.18, 1.52)	<0.001
Cirrhosis	9391(24.9)	2536(24.4)	0.97(0.92, 1.02)	0.01
Stroke	8822(23.4)	3647(35.0)	1.77(1.69, 1.85)	<0.001
GI bleeding	20988(55.6)	5787(55.6)	1.00(0.96, 1.04)	0.97
COPD	10410(27.6)	3408(32.7)	1.28(1.22, 1.34)	<0.001
Previous Hip fracture	6136(16.3)	2109(20.3)	1.31(1.24, 1.38)	<0.001
Osteoporosis	7477(19.8)	2240(21.5)	1.11(1.05, 1.17)	0.001
Dementia	2060(5.46)	1138(11.0)	2.13(1.97, 2.29)	<0.001
Previous herpes	1996(5.29)	562(5.40)	1.02(0.93, 1.13)	0.66
Previous respiratory failure	1344(3.56)	1216(11.7)	3.58(3.30, 3.88)	<0.001
Number of comorbidities				<0.001
≤5	18530(49.1)	4354(41.8)	1.00	
>5	19212(50.9)	6057(58.2)	1.34(1.28, 1.40)	

Data are presented as the number of subjects in each group with percentages given in parentheses or mean with standard deviation given in parentheses. Chi-square test, * Fisher-exact test and ^†^ Mann-Whitney U-test comparing subjects with and without death. ^&^ Urbanization level was categorized according to the population density of the residential area into 4 levels, with Level 1 the most urbanized and Level 4 the least urbanized. ^‡^ Other occupations included primarily retired, unemployed, or low income populations.

**Table 3:** Metrics for the random forest (RF) regression model for cost.

	MSE	MAE
All	0.666	0.491
Train	0.652	0.487
Test	0.754	0.513

MSE: mean squared error; MAE: mean absolute error.

**Table 4:** Metrics for the artificial neural network (ANN) regression model for cost.

	MSE	MAE
All	4.42948	1.85189
Train	4.43229	1.85185
Test	4.33447	1.85346

MSE: mean squared error; MAE: mean absolute error.

**Table 5:** The evaluation metrics of the random forest classification model for mortality.

	F_1_	Precision	Recall	AUROC	AUROC SE	AUROC 95% CI
All Subjects	0.780	0.843	0.817	0.861	0.002	0.857–0.864
Train Set	0.800	0.863	0.832	0.656	0.007	0.643–0.669
Test Set	0.672	0.702	0.743	0.656	0.007	0.643–0.669

**Table 6:** The evaluation metrics of the artificial neural network classification model for mortality.

	F1	Precision	Recall	AUROC	AUROC SE	AUROC 95% CI
All Subjects	0.661	0.717	0.640	0.685	0.003	0.680–0.691
Train Set	0.662	0.717	0.641	0.687	0.003	0.682–0.693
Test Set	0.658	0.715	0.634	0.675	0.007	0.662–0.688

**Table 7:** The separate cohorts that the models were evaluated on.

Cohort	Subjects	Subject Alive, *n* (%)	Subject Death, *n* (%)
Age < 70	13360	11513(86.2)	1847(13.8)
70 ≤ Age < 75	13372	10934(81.8)	2438(18.2)
75 ≤ Age < 80	11188	8579(76.7)	2609(23.3)
80 ≤ Age < 85	6852	4706(68.7)	2146(31.3)
85 ≤ Age < 90	2706	1681(62.1)	1025(37.9)
Age > 90	675	329(48.7)	346(51.3)

**Table 8:** The performance metrics of specifics cohorts on the RF model.

Cohort\Metric	F_1_	Precision	Recall
Age < 70	0.738	0.805	0.699
70 ≤ Age < 75	0.680	0.752	0.642
75 ≤ Age < 80	0.633	0.705	0.604
80 ≤ Age < 85	0.583	0.641	0.567
85 ≤ Age < 90	0.548	0.599	0.545
Age > 90	0.546	0.568	0.563

**Table 9:** The performance metrics of specifics cohorts on the ANN model.

Cohort\Metric	F_1_	Precision	Recall
Age < 70	0.818	0.849	0.868
70 ≤ Age < 75	0.779	0.839	0.835
75 ≤ Age < 80	0.714	0.808	0.787
80 ≤ Age < 85	0.613	0.722	0.705
85 ≤ Age < 90	0.545	0.666	0.643
Age > 90	0.430	0.645	0.530
